# Psychometric Properties of the Chinese Version of the 10-Item Ruminative Response Scale Among Undergraduates and Depressive Patients

**DOI:** 10.3389/fpsyt.2021.626859

**Published:** 2021-05-26

**Authors:** Jiayue He, Yalin Liu, Chang Cheng, Shulin Fang, Xiang Wang, Shuqiao Yao

**Affiliations:** ^1^Medical Psychological Center, The Second Xiangya Hospital, Central South University, Changsha, China; ^2^Medical Psychological Institute of Central South University, Central South University, Changsha, China; ^3^National Clinical Research Center for Mental Disorders, The Second Xiangya Hospital, Central South University, Changsha, China

**Keywords:** RRS-10, depression, rumination, factor structure, measurement invariance

## Abstract

**Objective:** Rumination is considered as a key process in the mechanism of depression. Assessing rumination is important for both research and clinical practice. The Ruminative Response Scale (RRS) is a widely-used instrument to measure rumination. This study aimed to examine the psychometric properties of the Chinese 10-item Ruminative Response Scale (RRS-10) in a large sample of Chinese undergraduates and depressive patients.

**Methods:** A total of 1,773 university students and 286 clinical patients with major depressive disorder finished the Chinese version of the RRS10, State Trait Anxiety Inventory (STAI), and Beck Depression Inventory (BDI). A confirmatory factor analysis (CFA) was performed to examine the two-factor structure (reflection and brooding) of the RRS-10. The correlations among RRS-10, STAI, and BDI were explored in two samples. In addition, the measurement invariance of the RRS-10 across gender, time, and groups with and without depressive symptoms were further investigated. The internal consistency and test-retest reliability were also evaluated.

**Results:** Confirmatory Factor Analysis revealed that the two-factor structure of RRS-10 fitted reasonably both in undergraduates (CFI = 0.933, TLI = 0.905, RMSEA = 0.071, SRMR = 0.035) and depressive patients (CFI = 0.941, TLI = 0.910, RMSEA = 0.077, SRMR = 0.057). The results of the multi-group confirmatory factor analysis supported the full strict invariance across genders and across groups (undergraduates and depressive patients). The full strong invariance over time was also supported by MGCFA. Besides, the RRS-10 showed acceptable internal consistency and good stability.

**Conclusions:** The RRS-10 has good reliability and validity in different samples and over time, which demonstrated that RRS-10 is a valid measurement instrument to assess rumination.

## Introduction

Rumination, defined as repetitive thoughts focusing on negative feelings and their causes, implications, and consequences is a method of coping with a negative mood. Individuals with a ruminative style of negative mood will focus their attention on their negative emotional state and ruminate about the causes of their depression and the features of its consequences so that they unable engage in some happy or neutral activities to get rid of their depression, thus prolonging the duration of depression. In 1987, Susan Nolen-Hoeksema first put forward the response style theory of depression (1). According to this theory, rumination is an important vulnerable factor for depression, which might aggravate and prolong depressive episodes (1–6). Researchers found that ruminative response could predict the severity of depression among clinical and non-clinical samples after 1 year (7). Longitudinal research has also revealed that even when controlling the most basic level of depression, rumination still has a significant effect on depressive symptoms (8–11). These results suggested that rumination is not inherently depressing, but can prolong an existing depressed mood.

On the basis of the response style theory, Nolen-Hoeksema et al. develop the Response Style Questionnaire (RSQ) to evaluate two different response styles of depression: rumination and distraction (12). The Ruminative Response Scale (RRS) was developed from the rumination subscale of the Response Style Questionnaire (RSQ). It has been revised over the years, leading to the current 22-item RRS. The RRS has shown good reliability (Cronbach α = 0.74–0.92, r_test−retest_ = 0.48–0.76) and good validity in the USA (13, 14), Japan (8), Korea (15), Dutch (16), Brazil (17), France (18), and Spain (19), and in clinical and non-clinical samples (20). The Chinese version of the RRS has been reported to be useful for assessing rumination in a large undergraduate sample (21).

In a principal component analysis study, Roberts et al. (22) determined that the RRS was composed of three dimensions: symptom-based rumination, introspection/self-isolation, and self-blame. Bagby et al. came up with the two-factor structure of RRS among clinical patients, including symptom-focused rumination and self-focused rumination (23). Treynor and his Colleagues found some of the RRS items overlapped with depression scale constructs, and were thus classified as depressed-symptom rumination items (24). Thus, previous studies have removed 12 depression-related items from the RRS and found that the structure of scale, consisting of the remaining 10 items, had two 5-item factors: *brooding* and *reflection* (24). The brooding dimension of the RRS-10 refers to “mood pondering” (e.g., “Why do I have problems other people don't have?”), whereas the reflection dimension describes cognitive (as opposed to emotive) reassessment of past and present events, feelings, and behaviors (e.g., “Go someplace alone to think about your feelings”). The original RRS10 two-factor model (Factor 1: brooding; Factor 2: reflection) which was investigated by Treynor has been confirmed in several studies (8, 14, 15, 21). However, there still have been some inconsistencies surrounding the two-factor model. For example, Arana et al. explored the fact that the two-factor structure only retained eight of the original items (excluding item 2 and item 9) (25). A confirmatory study found that the original two-factor model was not confirmed among a community sample (17). A number of studies in recent years have confirmed that different types of rumination have different effects on depression: brooding is a risk factor which may prolong or exacerbate depression, while reflection is a protective factor which does not prolong depression (11, 26). Thus, it is important to determine whether the two-factor structure of rumination is consistent among levels of depression (16). But there has been a relative paucity of research examining the factor structure of the RRS10 in depressive patients.

Another issue that needs to be further investigated is whether RRS-10 has the same structure in different groups and whether items have the same meaning for individuals in different groups. In the research of ruminative response, the comparison of the level of rumination between different groups is usually carried out without the test of measurement invariance. However, to make the scale effective and interpretable between group comparisons, it is necessary to prove whether it has measurement invariance (27). That is to say, measurement invariance is a prerequisite for the comparison of differences between groups (28). Measurement invariance is defined as “a given factorial defined construct has the same measurement parameters across two or more samples (i.e., the loadings, intercepts and residual matrix are equal among different groups)” (29). Without evidence of measurement invariance, it cannot be concluded that group difference in rumination reflected true differences between groups, as the difference may be due to the item bias of the scale (30).

According to the ruminative response style theory of depression, women have been shown to be more likely to ruminate about the causes of their mood than men in the face of depression (31), and it has been suggested that this difference in response styles could explain, at least in part, the gender disparity in adult depression (22, 32–34). To ensure such inter-group difference is valid, and not reflecting an artifact of the instrument, it is necessary to confirm consistency of meaning for the scale's items between groups (35). Thus, to compare gender differences in rumination, it is essential that gender invariance of the scale should be established (36). Previous study has demonstrated that the measurement invariance of the RRS-10 was acceptable across genders among an undergraduate sample (14), but the result was not generalized to clinical populations.

Moreover, to make valid score comparisons over time, it is important to assess whether scale items measure the same construct over time, a property known as longitudinal invariance (37). Although changes in rumination over time have been routinely investigated, few studies have explored the longitudinal invariance of the RRS-10 up to now (38). However, without prior testing of longitudinal measurement invariance, it is not possible to determine whether the time changes observed in a structure are due to real changes or to changes in structure or structural measurements over time (27).

In summary, measurement invariance of the RRS10 across gender was supported in a previous study, but the result was not generalized to clinical populations, whereas little research has examined measurement invariance of the RRS10 over time and between groups with and without depressive symptoms.

Thus, the aims of the present study were 3-fold. First, we tested the reliability of the RRS-10 in undergraduate and depressive patients. Second, we examined the two-factor model of the RRS-10 in the two samples. Third, we explored the measurement invariance of the RRS-10 across gender, time, and groups with and without depressive symptoms.

## Materials and Methods

### Participants and Procedure

Undergraduate participants were recruited from two Chinese universities in Hunan Province. The scale was completed in the classroom and the data were collected by well-trained psychology researchers. Students who had a history of mental disorder, history of neurological disorder, or intellectual disability were excluded. A total of 1,872 university students were surveyed, 10 of which were excluded due to a history of mental disorder and 89 of which were excluded due to missing data. The final student sample includes 1,773 participants (95% completion rate) who returned valid questionnaires. The mean age of the final sample were 18.86 years [standard deviation (SD) = 1.22], including 853 men with a mean age of 18.69 years (SD = 1.14) and 920 women with a mean age of 19.02 years (SD = 1.27). For test-retest reliability analysis and longitudinal invariance, a subgroup of 633 participants (343 men, 54%; and 290 women, 46%) completed the RRS-10 again 2 months later. They had a mean age of 18.39 years (SD = 0.90).

The clinical sample which consisted of depressive patients was recruited from the psychological clinic of Second Xiangya Hospital. Diagnosis was conducted independently by two psychiatrists using the Structured Clinical Interview for the DSM-IV-TR Axis I Disorders-Patient Edition. All the patients met the depression standard of the DSM-IV-TR. Exclusion criteria were (1) prior DSM-IV-TR Axis I disorder (except depressive disorder); (2) history of alcohol/substance abuse; (3) diagnosed neurological disorder; (4) intellectual disability. A total of 286 depressive patients provided complete data, including 126 men (44.1%) and 160 women (55.9%). They had a mean age of 23.25 (SD = 6.62).

Participants were told that the information in these scales would not be disclosed to anyone outside of the research team and all participants provided informed consent. The Ethics Committee of the Second Xiangya Hospital of Central South University (Code: 025) approved the study. The study was unpaid, and all the participants volunteered to complete the study.

### Measures

#### 10-item Ruminative Response Scale (RRS-10)

The 10-item RRS, which was part of the larger Response Styles Questionnaire, is a self-rating scale designed to assess thoughts and behaviors when people feel depressed (12). It has two subscales (Brooding and Reflection) and its items are graded on a scale from 1 (never) to 4 (always), with higher scores indicating a greater rumination tendency. The original RRS10 has been demonstrated to have high internal reliability and good test-retest reliability (15, 19, 20, 24). The Chinese version of RRS-10 was translated from English into Chinese by two psychologists, and then it was translated back into English by a bilingual translator with repeated revisions to ensure translation accuracy. No questionnaire item was removed or altered significantly during translation.

#### State-Trait Anxiety Inventory (STAI)

The widely used self-reported STAI (39) consists of State Anxiety Inventory (SAI) and Trait Anxiety Inventory (TAI) components for measuring the distinct concepts of state and trait anxiety (e.g., I feel nervous; I worry too much over something that really doesn't matter). Each component scale has 20 items answered on a 1–4 scale, with higher score indicating more severe anxiety symptoms. The STAI has high internal consistency (Cronbach's α: state anxiety = 0.89–0.95; trait anxiety = 0.89–0.92) and good test-retest reliability (*r* ranging from 0.62 to 0.96 for state anxiety and ranging from 0.84 to 0.98 for state anxiety over periods of 2 to 4 weeks) (40–42). The Chinese version of STAI also has good internal reliability (Cronbach's α: state anxiety = 0.91; trait anxiety = 0.92) and test-retest reliability (*r*: 0.91 for state anxiety and 0.76 for trait anxiety over 2 weeks) (43). In the present study, the STAI had good internal consistency (Cronbach's α: state anxiety = 0.89; trait anxiety = 0.84).

#### Beck Depression Inventory (BDI)

The BDI is a multiple-choice self-reporting 21-item scale (44) used primarily to assess the presence and severity of depressive symptoms in the prior 2 weeks in clinical and non-clinical populations (e.g., guilty feelings; loss of pleasure). Each question is answered on a 0–3-point scale of intensity. The BDI total score range is from 0 to 63 points, with higher scores indicating more severe symptoms. The Chinese version of the BDI has good reliability since the Cronbach's α was 0.94 for clinical samples and 0.88–0.94 for non-clinical samples (45). The BDI also exhibited good internal consistency (Cronbach's α = 0.85) in the present study. We used the BDI to assess the convergent validity of the RRS-10 with respect to conceptualization of rumination in relation to depressive mood or depressive symptoms.

### Data Analysis

To evaluate the reliability of the RRS-10, we calculated Cronbach's α values, mean inter-item correlations (MICs), split-half reliability, and test-retest reliability. Cronbach's α > 0.70 (>0.60 in some cases) was considered acceptable. MICs in the range of 0.10–0.40 were considered optimal.

To evaluate validity, we examined STAI and BDI score relationships with RRS-10 scores and its subscale. These analyses were conducted in IBM SPSS 20.0. The starting hypothesis was that there is a strong, positive correlation among RRS-10, BDI, and STAI. Moreover, to examine whether depression and anxiety were predicted by demographic variables (gender and age) and rumination, we performed multiple linear regression both in the undergraduate sample and the clinical sample, with the BDI total score and STAI total score as dependent variables, respectively.

Confirmatory factor analysis (CFA) with Weighted Least Squares Estimation was employed to determine the goodness of fit of the two-factor structure model of the RRS-10 in undergraduates and depressive patients to establish well-fitting baseline model. Model fit was assessed based on the comparative fit index (CFI), Tucker-Lewis index (TLI), standardized root mean square residual (SRMR), and root mean square error of approximation (RMSEA) with a 90% confidence interval (CI). The acceptable fit criteria applied were: CFI ≥ 0.90, TLI ≥ 0.90, SRMR ≤ 0.08, and RMSEA ≤ 0.08 (28, 46).

Multigroup CFA (MGCFA) was then used to examine the measurement invariance of the RRS-10 across gender, time, and groups. Four aspects of measurement invariance were tested (47, 48). First, configural invariance (Model 1) was examined to test the consistency latent variable constitution across groups. Second, weak invariance (Model 2) was examined to probe inter-group consistency of factor loading. Third, strong invariance (Model 3) was examined to test whether the intercepts of observed variables were equal across groups. Fourth, strict invariance (Model 4) was examined to test whether error variance was consistent across groups. Measurement invariance was inferred from changes in CFI (ΔCFI), TLI (ΔTLI), and RMSEA (ΔRMSEA) with the following acceptability criteria: ΔCFI ≤ 0.010, ΔTLI ≤ 0.010, and ΔRMSEA ≤ 0.015 (49). CFA and MGCFA were completed in Mplus 7.4.

## Results

### Reliability

Cronbach's α values, mean inter-item correlations, and split-half reliability for the RRS-10 were reported by sample in Table 1. In both samples, the Cronbach's α values were >0.8 for the whole scale and >0.7 for each dimension. All mean MICs were between 0.310 and 0.400. Split-half reliability was slightly higher in the clinical sample (0.744–0.763) than in the undergraduate sample (0.706–0.729). Good test-retest reliability for the full scale and each dimension were confirmed in the undergraduate sample (Table 1).

**Table 1:** Cronbach's α values, mean inter-item correlations, split-half reliability, and test-retest reliability of the RRS-10 and its two dimensions by sample.

	**Undergraduate sample**				**Clinical sample**		
	**Cronbach's α**	**MIC**	**Split-half coefficient**	**Test-retest coefficient**	**Cronbach's α**	**MIC**	**Split-half coefficient**
RRS-10	0.819	0.310	0.729	0.895	0.831	0.310	0.763
Brooding	0.719	0.337	0.706	0.660	0.709	0.400	0.747
Reflection	0.743	0.367	0.720	0.776	0.768	0.337	0.744

### Convergent Validity

As shown in Table 2, Pearson analyses demonstrated very significant (*p* <0.01) positive correlation coefficients among RRS-10 total scale, brooding subscale, reflection subscale, BDI, and STAI scores in both undergraduate sample and clinical sample. These direct correlations indicated good convergent validity of the RRS-10 with depression and anxiety scales.

**Table 2:** Correlations among STAI, BDI, and RRS-10 scores.

	**Undergraduate sample**				**Clinical sample**			
	**Brooding**	**Reflection**	**RRS-10**	**STAI**	**Brooding**	**Reflection**	**RRS-10**	**STAI**
Reflection	0.521^**^				0.593^**^			
RRS-10	0.849^**^	0.894^**^			0.848^**^	0.711^**^		
STAI	0.287^**^	0.112^**^	0.218^**^		0.435^**^	0.217^**^	0.640^**^	
BDI	0.399^**^	0.177^**^	0.285^**^	0.610^**^	0.424^**^	0.241^**^	0.638^**^	0.813^**^

** *p < 0.01*.

We then examined the joint contribution of RRS-10 and demographic variables to predict depressive and anxiety scores through a series of multiple regression analyses. The results in Table 3 showed that gender and brooding subscale scores were significant predictors of BDI total score in the undergraduate sample, whereas gender, age, brooding subscale score, and reflection subscale scores were significant predictors of STAI total score in the undergraduate sample. Only brooding subscale score was a significant predictor of BDI total score and of STAI total score in the clinical sample (Table 3).

**Table 3:** Multiple regression analyses with BDI total score (above) and STAI total score (below) as the dependent variable.

	**Undergraduate sample**				**Clinical sample**			
	**ß**	**95%CI**	***t***	***P***	**ß**	**95%CI**	***t***	***P***
*BDI*								
Gender	0.071	0.387, 1.678	3.137	**0.002**	0.091	−0.572, 5.357	1.589	0.113
Age	0.041	−0.006, 0.153	1.807	0.071	0.074	−0.078, 0.374	1.287	0.199
Brooding	0.325	0.796, 1.097	12.358	**<** **0.001**	0.424	1.098, 2.187	5.942	** <0.001**
Reflection	0.006	−0.112, 0.142	0.236	0.813	0.003	−0.544, 0.566	0.040	0.968
	*F* = 57.013, ***p*** ** <0.01**, *R*^2^ = 0.116				*F* = 15.190, ***p*** ** <0.01**, *R*^2^ = 0.195			
*STAI*								
Gender	0.130	2.739, 5.652	5.651	** <0.001**	0.056	−2.694, 8.067	0.983	0.326
Age	0.074	0.115, 0.473	3.229	**0.001**	0.038	−0.255, 5.151	0.666	0.506
Brooding	0.313	1.652, 2.337	11.411	** <0.001**	0.470	2.302, 4.275	6.566	** <0.001**
Reflection	−0.059	−0.625, 0.030	−2.159	**0.031**	−0.062	−1.420, 0.544	−0.878	0.381
	*F* = 50.250, ***p*** ** <0.01**, *R*^2^ = 0.106				*F* = 15.806, ***p*** **<** **0.01**, *R*^2^ = 0.198			

*Significant p-values are bolded*.

### CFA

All fit indices of the two-factor model reached our acceptability criteria in the undergraduate sample and clinical sample (Undergraduate sample: CFI = 0.933, TLI = 0.905, RMSEA = 0.071, SRMR = 0.035; Clinical sample: CFI = 0.941, TLI = 0.910, RMSEA = 0.077, SRMR = 0.057) (Table 4). Hence, CFA confirmation of the two-factor structure of the RRS-10 indicated that this model could be used as a baseline model for measurement invariance testing. The factor loadings of each item were shown in Table 5 (The Chinese items were showed in Supplementary Material). All items factor loadings were 0.35 or greater.

**Table 4:** Goodness of fit indexes for the two-factor model of the RRS-10.

	**χ^2^**	**df**	**CFI**	**TLI**	**SRMR**	**RMSEA**
Undergraduate sample	4323.7	45	0.933	0.905	0.035	0.071
Clinical sample	77.264	30	0.941	0.910	0.057	0.077

*χ^2^, Chi-square; df, degrees of freedom; CFI, Comparative Fit Index; SRMR, Standardized Root Mean Square Residual; RMSEA, Root Mean Square Error of Approximation*.

**Table 5:** The factor loadings of each item in RRS-10.

**RRS-10 item**	**Undergraduate sample**	**Clinical sample**
Think “What am I doing to deserve this?”	0.401	0.460
Analyze recent events to try to understand why you are depressed.	0.664	0.686
Think “Why do I always react this way?”	0.601	0.369
Go away by yourself and think about why you feel this way.	0.513	0.729
Write down what you are thinking and analyze it.	0.565	0.723
Think about a recent situation, wishing it had gone better.	0.574	0.585
Think “Why do I have problems other people don't have?”	0.682	0.724
Think “Why can't I handle things better?”	0.503	0.550
Analyze your personality to try to understand why you are depressed.	0.625	0.743
Go someplace alone to think about your feelings.	0.643	0.530

### Measurement Invariance Across Gender

Based on the two-factor model of RRS-10, we proceeded with subsequent measurement invariance testing. Our first model specified configural invariance, meaning that the same factor structure was estimated for women and men; no inter-group constraints were placed on the parameter estimates. All goodness of fit indices obtained met the requirements of configural invariance (Table 6). Thus, configural invariance was established and the model was used as a baseline model for weak invariance (Model 6) analysis, in which factor loading was equalized across the groups. All goodness of fit indices met the requirements of weak invariance (ΔCFI = 0.001, ΔTLI = −0.008, and ΔRMSEA = 0.003). Thus, weak invariance was established between males and females, and strong invariance (Model 3) was examined with equal intercepts across genders. All requirements for goodness of fit indices for the strong invariance test were met (ΔCFI = 0.002, ΔTLI = −0.004, and ΔRMSEA = 0.002). Finally, for strict invariance testing (Model 4), the additional constraint of equal error variance across the two groups was added. All criteria for indices of strict invariance were met (ΔCFI = 0.007, ΔTLI = 0.000, and ΔRMSEA = 0.002), as shown in Table 6, establishing strict invariance for the undergraduate sample. Together, these results support the configural, metric, scalar, and strict invariance of the re-specified two-factor model of the RRS-10 across genders in our undergraduate sample.

**Table 6:** Measurement invariance of the RRS-10 across gender.

**Model**	**χ^2^**	**df**	**CFI**	**TLI**	**SRMR**	**RMSEA**	**ΔCFI**	**ΔTLI**	**ΔRMSEA**
*Undergraduate sample*									
Model 1	215.461	60	0.951	0.926	0.037	0.053	–	–	–
Model 2	224.761	68	0.950	0.934	0.039	0.050	0.001	−0.008	0.003
Model 3	241.059	76	0.948	0.938	0.040	0.048	0.002	−0.004	0.002
Model 4	272.072	86	0.941	0.938	0.045	0.048	0.007	0.000	0.002
*Clinical sample*									
Model 1	88.136	53	0.949	0.913	0.057	0.069	–	–	–
Model 2	97.831	61	0.946	0.921	0.063	0.066	0.003	−0.008	0.003
Model 3	111.083	69	0.939	0.920	0.068	0.066	0.007	0.001	0.000
Model 4	136.978	80	0.917	0.906	0.071	0.072	0.022	0.014	−0.006

*Model 1: morphological invariance; Model 2: metric invariance; Model 3: strong invariance; Model 4: strict invariance*.

In our clinical sample, the baseline models were deemed suitable for representing the data for males and for females (fit indices reported in Table 6), providing evidence in support of configural invariance. Furthermore, the changes observed in CFI (<0.010), TLI (<0.010), and RMSEA (<0.015) supported both weak equivalence and strong equivalence of the RRS-10 (Table 6). However, strict invariance was not supported since ΔCFI and ΔTLI >0.01 (ΔCFI = 0.022, ΔTLI = 0.014).

### Measurement Invariance Across Time

Model fitting indexes for configural and weak invariance models met our measurement invariance requirements, and the changes in CFI, TLI, and RMSEA values supported weak and strong equivalence of the RRS-10 across the two testing time points (Table 7). Thus, the measurement invariance of the RRS-10 across time was established.

**Table 7:** Measurement invariance of the RRS-10 across time and across samples with and without depressive symptoms.

**Model**	**χ2**	***df***	**CFI**	**TLI**	**SRMR**	**RMSEA**	**ΔCFI**	**ΔTLI**	**ΔRMSEA**
*Measurement invariance across time*									
Model 1	226.929	64	0.932	0.904	0.045	0.063	–	–	–
Model 2	255.836	74	0.924	0.907	0.048	0.062	0.008	−0.003	0.001
Model 3	279.870	82	0.917	0.909	0.050	0.062	0.007	−0.002	0.000
Model 4	279.068	90	0.921	0.921	0.052	0.058	−0.004	−0.012	0.004
*Measurement invariance across samples with and without depressive symptoms*									
Model 1	340.632	64	0.939	0.914	0.041	0.065	–	–	–
Model 2	372.675	72	0.934	0.917	0.049	0.064	0.005	−0.003	0.001
Model 3	392.447	74	0.930	0.915	0.056	0.065	0.004	0.002	−0.001
Model 4	385.503	68	0.930	0.907	0.059	0.068	0.000	0.008	−0.003

*Model 1: morphological invariance; Model 2: metric invariance; Model 3: strong invariance; Model 4: strict invariance*.

### Measurement Invariance Across Clinical and Non-clinical Samples

The model fitting indexes for configural and weak invariance models met our measurement invariance requirements, and the changes in TLI, CFI and RMSEA values supported weak, strong, and strict equivalence of the RRS-10 across our non-clinical (undergraduate sample) and clinical samples (depressive patients) shown in Table 7. These results indicated that the RRS-10 exhibited good measurement invariance across clinical (i.e., depressive patients) and non-clinical samples.

## Discussion

The current study aimed to examine the reliability and validity of the Chinese RRS-10 in clinical and non-clinical samples. The high internal consistency and test-retest reliability values were obtained in two samples. Then, a CFA was conducted, supporting a similar two-factor structure as that established in previous studies (8, 14, 15, 21, 24). Measurement invariance of the Chinese RRS-10 were well-established across gender, time, and groups with and without depressive symptoms. To our knowledge, this was the first research to explore the measurement invariance across times and different groups (clinical and non-clinical). The present results showed excellent reliability and validity of the RRS-10 in the clinical and non-clinical groups.

For the reliability analysis of the RRS-10, all Cronbach's α coefficients, in both the undergraduate sample and clinical sample, reached acceptable standards (α > 0.70). These results were consistent with previous studies, which reported the internal reliability from 0.74 to 0.92 (8, 13–19). All the mean inter-item coefficients were between 0.10 and 0.40 both in the undergraduate sample and clinical sample and the high test-retest values also indicated good reliability of the RRS-10.

According to the convergent validity, moderate but positive correlations were found between rumination (RRS-10 and its subscales) and psychiatric symptoms (depression and anxiety) in clinical and non-clinical groups, which demonstrate that individuals who had more ruminative thinking seemed to have greater depressive and anxiety symptoms. Previous research has shown a strong link between rumination and psychiatric illness, especially depression (50, 51). Lam et al. (52) found that, in a non-clinical group, RRS scores predicted a more predominant ruminative response style. A clinical research also found a strong association between the RRS score and the duration and severity of depressive episodes in patients with depression.

Furthermore, in multiple regression analyses, we found that the brooding subscale of the RRS-10 was a significant predictor of depression and anxiety symptoms in both undergraduates and clinical samples. The concepts of brooding and reflection in the context of the RRS represent two different types of rumination, with the former encompassing repeated, passive attending to one's own negative emotions and evaluating one's own status and goals harshly. Reflection, on the other hand, involves one's efforts to solve problems and, thereby, alleviate his or her symptoms of depression and anxiety. Brooding is associated with increased negativity bias and negative coping styles, while reflective rumination has a less clear relationship with negative outcomes (53). A meta-analysis indicated that brooding had a moderate effect size for suicidal ideation and history of suicide attempt, but reflection was only associated with suicidal ideation (54). Ricarte et al. also found that anxiety and brooding were positively correlated even after controlling for depression scores (55). Meanwhile, our findings that reflection subscores were associated with STAI scores, but not BDI scores, suggests that reflection may play an important role in anxiety disorders rather than depressive illnesses (56, 57).

Our CFA confirmation of the goodness of fit of the two-factor structure of the Chinese RRS-10 in our undergraduate and clinical samples were consistent with previous RRS factor analysis studies demonstrating a well-stabilized two-factor structure (19). Based on this result, we were comfortable employing the two-factor structure of the RRS-10 as a baseline model for examining measurement invariance.

Importantly, researchers' ability to compare groups in a valid way is dependent upon measurement invariance (30). The present study examined the measurement invariance of the RRS-10 across gender, time, and groups (clinical and non-clinical). Our MGCFA confirmed good morphological, weak, strong, and strict invariance of the Chinese RRS-10 across gender in undergraduate samples, which was consistent with previous studies (14, 21). The configural invariance was supported, which indicated that rumination was conceptualized similarly in women and men which was reflected by two factors measuring brooding and reflection. Besides, there was support for weak invariance, which means that the units of measurement are equal in men and women, that is, the items and potential factors of the scale have the same meaning in men and women (58). Moreover, the present establishment of strong invariance indicated that inter-gender group differences in scores could be interpreted as reflecting true group differences in latent variables, which provided the same reference point between men and women. Intergroup comparisons were meaningful only if the units and reference points are the same. Therefore, it is the premise to compare the latent mean that the weak equivalence and the strong equivalence are satisfied (58). Finally, the strict invariance was supported in women and men which reflected the cross-group difference of latent variable variation. In clinical samples, the strict equivalence was not supported. But the residual equivalence is the most strict equivalence limit and it is not necessary for most research (59). In summary, the results of this study confirmed that the Chinese RRS-10 has strong equivalence, indicating that the scale is effective and interpretable between gender groups.

Regarding measurement invariance over time, our results supported the conclusion that the Chinese RRS-10 had configural, weak, and strong invariance between an initial test and a re-test 2 months later, at least for general population individuals. This confirmation of longitudinal invariance indicated that researchers could be confident that changes in RRS-10 scores over time reflect real changes in rumination over time, rather than an artifact produced by composition instability. Because longitudinal invariance was assessed over a relatively short 2-month time interval, it is impossible to draw conclusions about the stability and structural invariance of RRS-10 over much longer intervals, such as several years or decades. Longer-term research is needed to further verify the longitudinal invariance of RRS-10 over longer periods of time.

The present research also supports the conclusion that the RRS-10 has configural, weak, strong, and strict measurement invariance between non-clinical (undergraduates) and clinical (depressive) samples. These results indicate that the form of latent variables in the RRS-10 is consistent between healthy adults and depressive patients, with equivalent factor loading, intercepts, and error variances of each item. This establishment of scale equivalence allows the inferences that the RRS-10 has the same reference point between clinical and non-clinical populations, and that the relationship between the scale's observation indicators and potential individual characteristics have the same meaning between general population and depressive groups.

Several limitations of our study should be acknowledged. First, the data were obtained principally from self-report measures, which are by nature subjective. Second, we did not control for socioeconomic and demographic variables (e.g., family income, religion, social relationships), which are associated with ruminative response and could affect the results of the RRS-10. Third, the samples only included undergraduate and depressive patients, thus limiting the generalizability of the results. Fourth, the level of rumination was different across cultures, thus, the measurement invariance of the RRS-10 across different cultures could be tested in the future.

## Conclusion

The RRS-10 has good psychometric characteristics and measurement invariance across gender, time, and populations with and without depressive symptoms. The present results support the conclusion that the RRS-10 is a valid and reliable self-reported instrument for examining rumination, in relation to depressed mood, in Chinese adults and in patients with depressive symptoms.

## Data Availability Statement

The raw data supporting the conclusions of this article will be made available by the authors, without undue reservation.

## Ethics Statement

The studies involving human participants were reviewed and approved by the Ethics Committee of the Second Xiangya Hospital of Central South University. The patients/participants provided their written informed consent to participate in this study. Written informed consent was obtained from the individual(s) for the publication of any potentially identifiable images or data included in this article.

## Author Contributions

SY supervised the study. JH and YL performed, collected, and wrote the paper. CC and SF contributed to the analysis. XW revised the paper. All co-authors revised and approved the version to be published.

## Conflict of Interest

The authors declare that the research was conducted in the absence of any commercial or financial relationships that could be construed as a potential conflict of interest.
