# Supplementary material for: Psychometric Properties of the Chinese Version of the 10-Item Ruminative Response Scale Among Undergraduates and Depressive Patients
Source: Front Psychiatry. 2021 May 26;12:626859. doi: 10.3389/fpsyt.2021.626859 (PMC8187760; doi:10.3389/fpsyt.2021.626859)
Supplement: Supplementary file 1 [file Table_1.DOCX]

**冗思反应量表**

人们在感到忧伤**时**会去想或者去做许多不同的事情。请阅读以下各项，看看当你感到忧伤、沮丧时是否有这些想法和行动及其程度，请在右边相应的数字上打勾。

1分：从来没有； 2分：有时； 3分：经常； 4分：总是发生

请记住依据你**通常的做法**作答，而不是想当然自己会怎样做。

| **内容** | **从来**  **没有** | **有时** | **经常** | **总是**  **如此** |
| --- | --- | --- | --- | --- |
| 1．我究竟做了什么要遭如此报应 | **1** | **2** | **3** | **4** |
| 2．分析新近发生的事情试图找到心情沮丧的原因 | **1** | **2** | **3** | **4** |
| 3．想到“我为什么总是有这种反应” | **1** | **2** | **3** | **4** |
| 4．一个人走开，思考自己为什么会有这种感觉 | **1** | **2** | **3** | **4** |
| 5．记录你自己的想法并做分析 | **1** | **2** | **3** | **4** |
| 6．回想新近的情境，希望情形已经好转 | **1** | **2** | **3** | **4** |
| 7．想到“为什么我有这样问题而别人没有。” | **1** | **2** | **3** | **4** |
| 8．想到“我为什么不能把事情做得更好一点﹖” | **1** | **2** | **3** | **4** |
| 9．分析自己的性格试图找到沮丧的原因 | **1** | **2** | **3** | **4** |
| 10．独自去某个地方考虑自己的感受 | **1** | **2** | **3** | **4** |
